# Milled Crown on Post Crack Tooth Syndrome as the Denture Abutment Tooth: A Case Report

**DOI:** 10.7759/cureus.39380

**Published:** 2023-05-23

**Authors:** Sarah Sabrina Mahalil Amin, John Chong Keat Hon, Muhammad Annurdin Sabarudin, Wan Nor Syariza Wan Ali, Nusima Mohamed

**Affiliations:** 1 Faculty of Dentistry, Universiti Sains Islam Malaysia, Kuala Lumpur, MYS

**Keywords:** extracoronally splint, removable partial denture, abutment tooth, milled crown, crack tooth syndrome

## Abstract

This clinical case describes using a milled crown on a cracked tooth as the abutment for a removable partial denture. A 63-year-old male patient diagnosed with lipoma undergoing chemotherapy and radiotherapy presented with symptomatic crack tooth syndrome on tooth 36 and partial edentulism. Conservative treatment using a molar band to extracoronally splint the tooth was conducted to determine the prognosis of the crack line. A lower partial cobalt-chromium denture was constructed by incorporating the milled crown of tooth 36 as the abutment. After six months of follow-up, there were no crack tooth symptoms, and regular review was adopted to monitor the tooth. The construction of a milled crown of a cracked tooth presented good and promising clinical outcomes in preserving tooth vitality and preventing crack propagation in partially dentate dentition for the long term.

## Introduction

Crack tooth syndrome (CTS) is a condition of incomplete fracture of a vital tooth involving dentine, which sometimes extends to the pulp, previously known as cuspal fracture odontalgia [1]. This condition usually affects posterior teeth; normally, patients with a history of pain on biting tend to avoid consuming the complaint site of CTS. The consumption of cold foods and drinks may also elicit pain. Patients may also exhibit signs and symptoms similar to irreversible pulpitis if the crack has propagated into the dental pulp area.

CTS has been associated with four major causative factors, according to Lynch et al., such as restorative procedures, occlusal factors, developmental conditions, and miscellaneous factors [1]. Restorative factors may include excessive removal of tooth structure during cavity preparation or placement of restorative material in bulk, inducing stress and weakening the remaining tooth structure. Besides, trauma from occlusion when biting hard food using excessive force may also lead to cuspal fracture.

Treatment of CTS depends on the site, direction, and degree of crack [2]. Splinting or immobilising the crack segment is the first line of treatment, including the placement of copper rings or stainless-steel bands. It is suggested to be clinically effective, minimally invasive, acceptable for patients, and cost-effective immediate treatment for CTS [3]. Root canal treatment is indicated if thermal sensitivity persists after immediate CTS therapy. Direct or indirect restoration, which may or may not provide usual coverage, has been proposed to treat the cracked tooth. It has been recommended by Guthrie et al. that full coverage indirect restoration, such as the crown, is the most appropriate restoration to manage CTS cases [3]. Full coverage restoration will minimise independent movement of the tooth, minimising stress, which will prevent the crack. This case report highlights the management of CTS using milled crowns incorporated with cobalt-chromium dentures.

## Case presentation

A 63-year-old male was referred from Outpatient Dental Polyclinic, Universiti Sains Islam Malaysia (USIM), for periodontal management at the Undergraduate Dental Clinic. Initially, the patient complained of sensitivity at the lower left molar tooth upon cold and hot food/drinks. Upon chewing hard food such as peanuts, he also experienced slight discomfort in that tooth. Thus, he avoided chewing on that side. The patient was a regular dental attendee, brushing his teeth five times daily with a medium-bristled toothbrush and non-fluoridated toothpaste. The patient had a medical history of hospitalisation due to lipoma removal of his left thigh in May 2017 and an oesophagectomy in January 2021. Apart from that, he completed multiple radiotherapy sessions in 2020 and implanted a chemoport in his right chest. His medical condition is currently stable, and he has undergone routine follow-up at Hospital Kuala Lumpur. He does not smoke and has no parafunctional habits. The patient was motivated and keen on dental treatment to improve his oral health.

Clinically, he presented with a generalised inflamed and reddish gingiva with a soft consistency of thin flat gingival phenotype. Multiple dental caries were noted on the upper anterior teeth and generalised cervical abrasion due to incorrect toothbrushing technique. Additionally, he also presented with a partially dentate lower arch, classified as Kennedy Class III modification 1.

**Figure 1:**
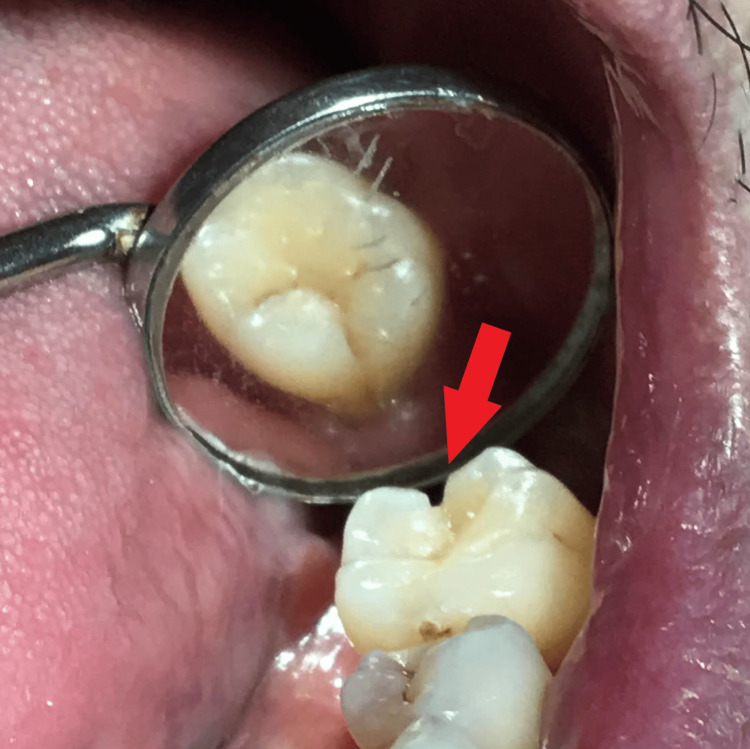
Pre-treatment clinical photo prior to molar band placement showing extension crack line at distoocclusal area (red arrow).

**Figure 2:**
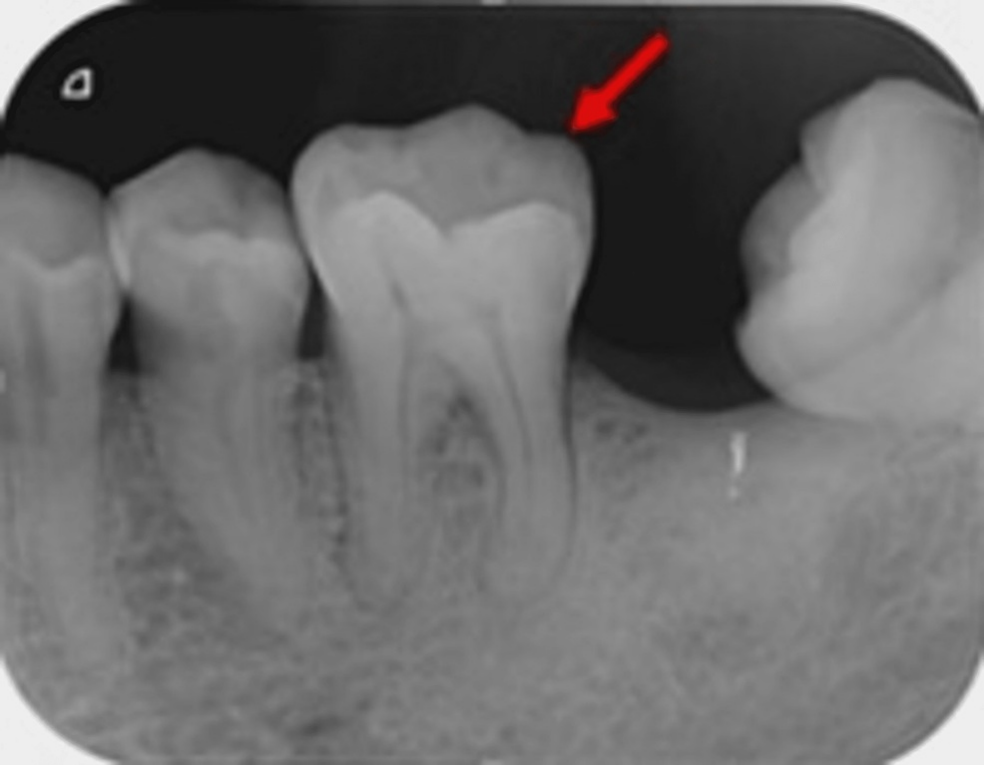
Periapical radiograph showing widening of periodontal ligament of tooth 36 and crack line noticeable at distoocclusal area (red arrow).

Upon clinical examination, tooth 36 presented with abrasion at the cervical area and a visible crack line from the distal marginal ridge to the equigingival level (Figure 1). The patient experienced slight discomfort at biting upon the bite test, and the tooth responded to the sensibility test. Radiographically, an intraoral periapical radiograph of tooth 36 was taken to assess the crack line and its periapical condition (Figure 2).

The patient was diagnosed with reversible pulpitis associated with CTS on tooth 36 as in Figures 1, 2, localised periodontitis stage III grade B without modifying risk factors, multiple carious teeth, and non-carious tooth surface loss due to his improper oral hygiene practice.

**Figure 3:**
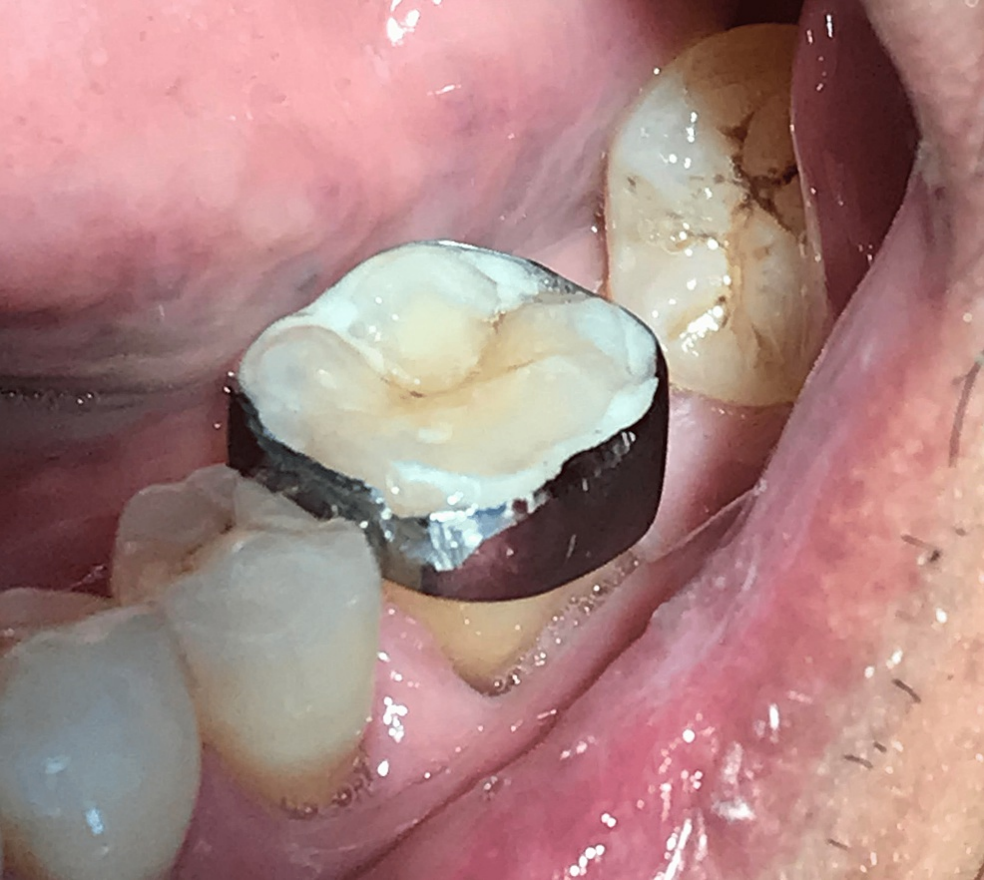
Placement of molar band #LL29 on cracked tooth syndrome tooth 36.

The first-line management was done with extracoronal splinting using a molar band placed on the tooth (Figure 3). Post-operative instructions were given, especially emphasising to avoid chewing hard food on that region. Apart from that, scaling, root surface debridement and restoration of multiple carious and tooth surface loss teeth were done. The construction of a full metal milled crown on the cracked tooth has also been incorporated with a lower partial denture to restore his dentition.

After three months of reviewing the signs and symptoms of tooth 36 concurrent with other restorative treatments, the tooth resolved its symptoms. During the re-evaluation visit, the patient’s oral hygiene was improved and revealed a healthy periodontium and teeth restoration was intact. At definitive management, a pre-prosthetic assessment was done. Occlusal analysis revealed that he has bilateral group function upon lateral excursions and incisal guidance upon protrusion movement. Considering the vitality of the tooth, the full metal milled crown was planned, as it is more conservative. Note that a mesial rest seat was incorporated into the prosthetic crown to place occlusal rest to support the cobalt-chromium partial denture. A buccal undercut was also incorporated to facilitate the C-clasp placement for partial denture retention.

**Figure 4:**
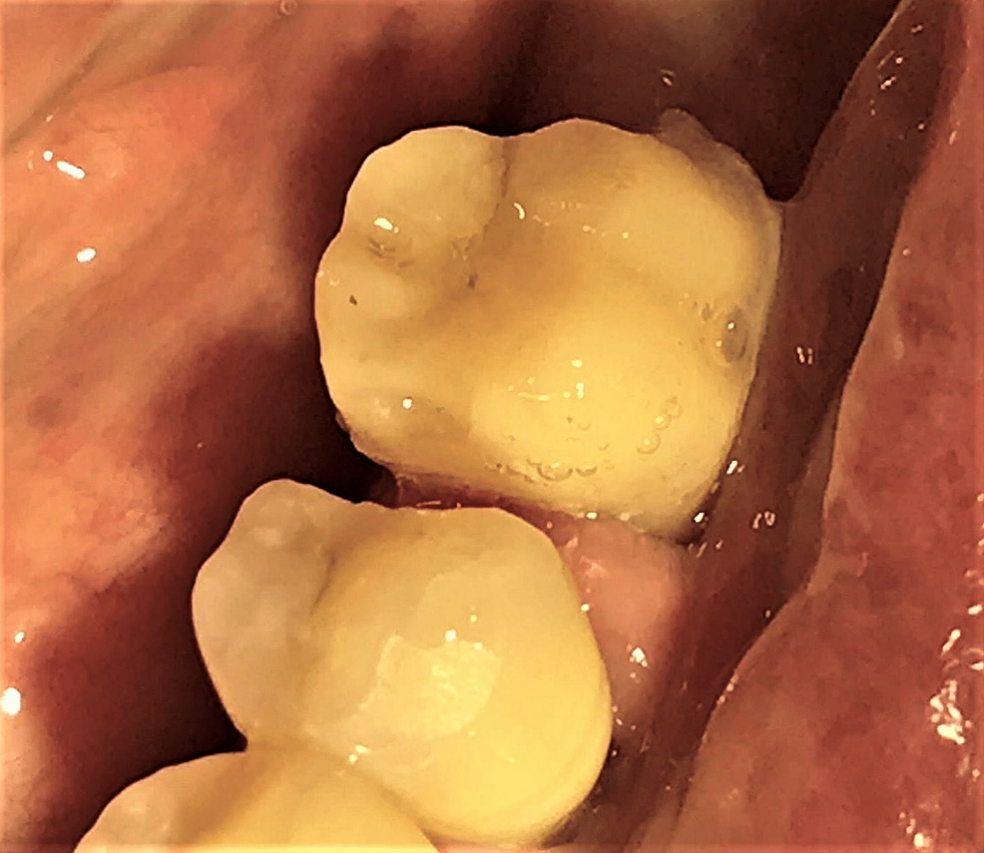
Final tooth preparation of tooth 36.

The tooth margin was prepared supragingivally on buccal and lingual surfaces, but the distal margin was prepared apically beyond the crack line to remove the visible crack line (Figure 4).

**Figure 5:**
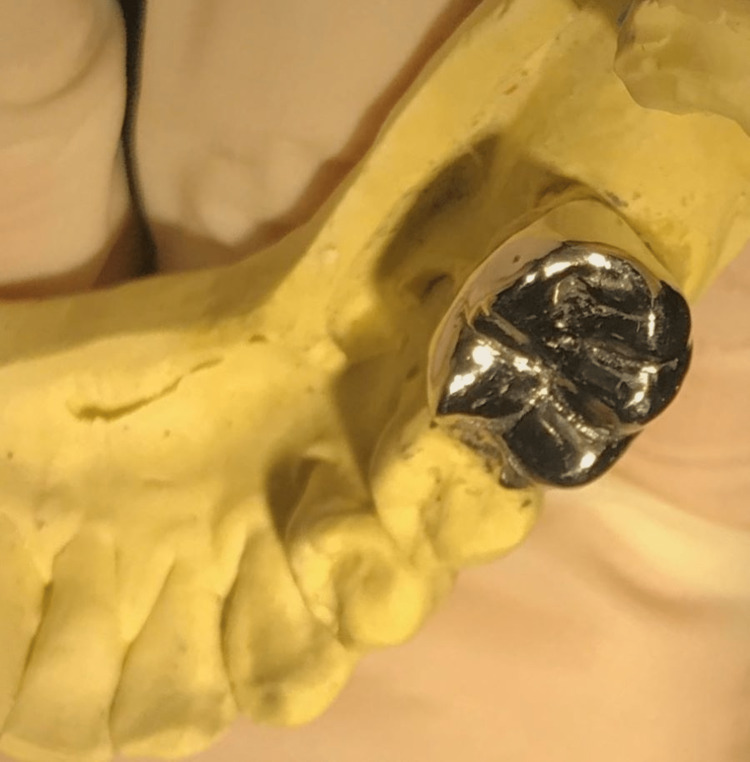
Full metal crown on master cast.

**Figure 6:**
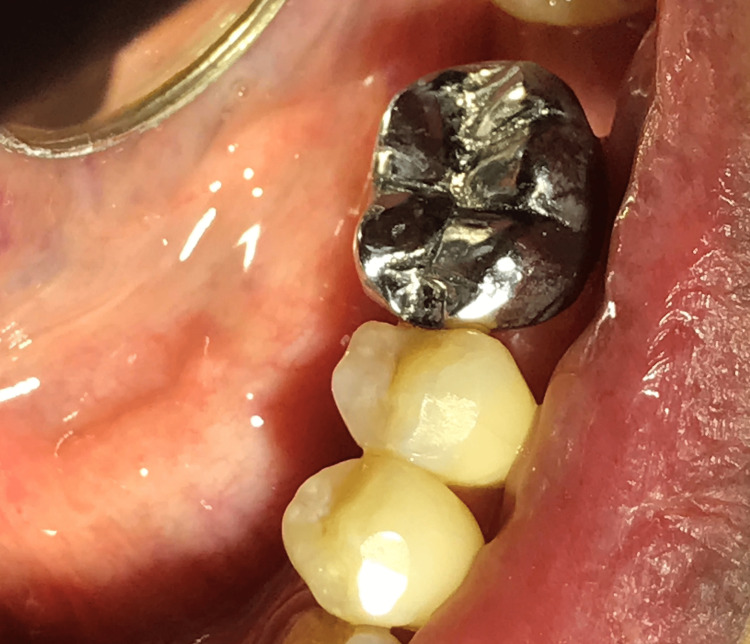
Full metal crown cemented on tooth 36 with mesial rest seat.

A temporary crown was placed on tooth 36 using bis-acryl composite for diagnostic purposes prior to the construction of the definitive crown. During the one-month follow-up visit, the patient claimed no symptoms arose from the tooth and temporary crown. Therefore, the final impression was taken using light and medium body polyvinyl siloxane material and sent to the lab to construct a full metal crown (Figures 5, 6).

**Figure 7:**
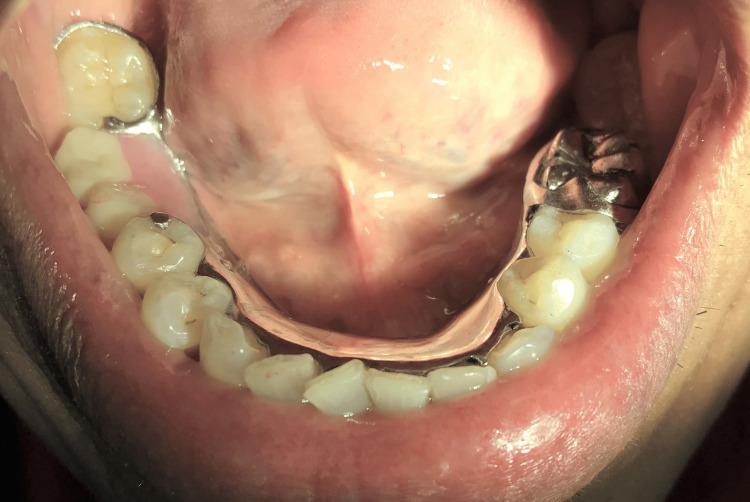
Milled crown with lower cobalt-chrome partial denture.

The crown was polished prior to cementation. The cobalt-chromium partial denture was constructed for the lower arch after the cementation of the crown (Figure 7).

Post-operatively, he was advised to adhere to oral and denture hygiene instructions, such as using floss to clean the crown area and cleaning and removing dentures at night before bed. He was reviewed until up to six months, and the patient had no complaints on the full metal crown of 36 as well as the lower partial cobalt-chromium denture.

## Discussion

The patient had complained that his lower left molar tooth had been diagnosed with CTS, which was later reinforced with the molar band to reduce the crack propagation and needed to be reviewed for its signs and symptoms. After a few review visits concurrent with other treatments, the tooth has resolved its symptoms. A full metal crown was chosen after discussing with the patient to be conservative and protect the pulp due to its history of cracked teeth. The milled crown acts its purposes to prevent crack propagation, protect the tooth and act as an abutment for the denture. One study discovered that metal crowns had a five-year survival rate of 80% [4], and the overall longevity of cracked teeth were also presented to be increased with the placement of full coverage crowns [5]. Glass ionomer cement was chosen for sustainable fluoride release for the crack tooth [6].

The patient was concerned regarding the Islamic jurisprudence of wearing a fixed prosthesis in his mouth. Islam permits the crowning method if it is for functional purposes and not solely for aesthetics. Referring to Imam al-Syafie’s scholars (an-Nawawi 1997), teeth are not a part of the body that needs to be washed (for wudhu’) as it is in our body [7,8].

Another concern of the patient was the elongation of the upper right molar teeth due to the missing opposing teeth, which led to overeruption. Construction of a lower partial cobalt-chromium denture was proposed to prevent any further overeruption of the upper molar teeth and improve his masticatory function. Kennedy Class III modification 1 cobalt-chromium removable partial denture (RPD) was selected to improve the occlusal function and also due to the high survival rate, indicating at least a 75% survival rate for over five years [9].

In this case, tooth 36 is the abutment for the lower RPD. The full metal crown has been designed with an occlusal rest seat [10] and a buccal undercut to facilitate the placement of a C-clasp from the metal framework. It is interesting to highlight that even though the tooth presented with CTS prior to the construction of the crown, it is still being utilised as the abutment for cobalt-chromium denture due to the unavailability of a posterior abutment with missing tooth 37 and mesially tilted 38. The best treatment option would be to implant placement to replace the missing teeth to avoid the use of tooth 36 as the abutment for the lower partial denture. However, in this case, the prognosis of the treatment is considered good since the diagnostic evaluations of crack symptoms and progression had been conducted by placing a stainless-steel molar band for three months and placing a temporary crown for one month prior to the construction of a definitive crown [11]. The six-month review visit was also uneventful.

## Conclusions

Full coverage crown on CTS minimises independent movement of the tooth. Hence, it minimises stress, which will relay the crack and improve patient mastication.
